# Supplementary material for: The force loading rate drives cell mechanosensing through both reinforcement and cytoskeletal softening
Source: Nat Commun. 2021 Jul 9;12:4229. doi: 10.1038/s41467-021-24383-3 (PMC8270983; doi:10.1038/s41467-021-24383-3)
Supplement: Supplementary file 1 — Supplementary Information [file 41467_2021_24383_MOESM1_ESM.pdf]

Supplementary information to

The force loading rate drives cell mechanosensing through both reinforcement and cytoskeletal softening.

Supplementary figures

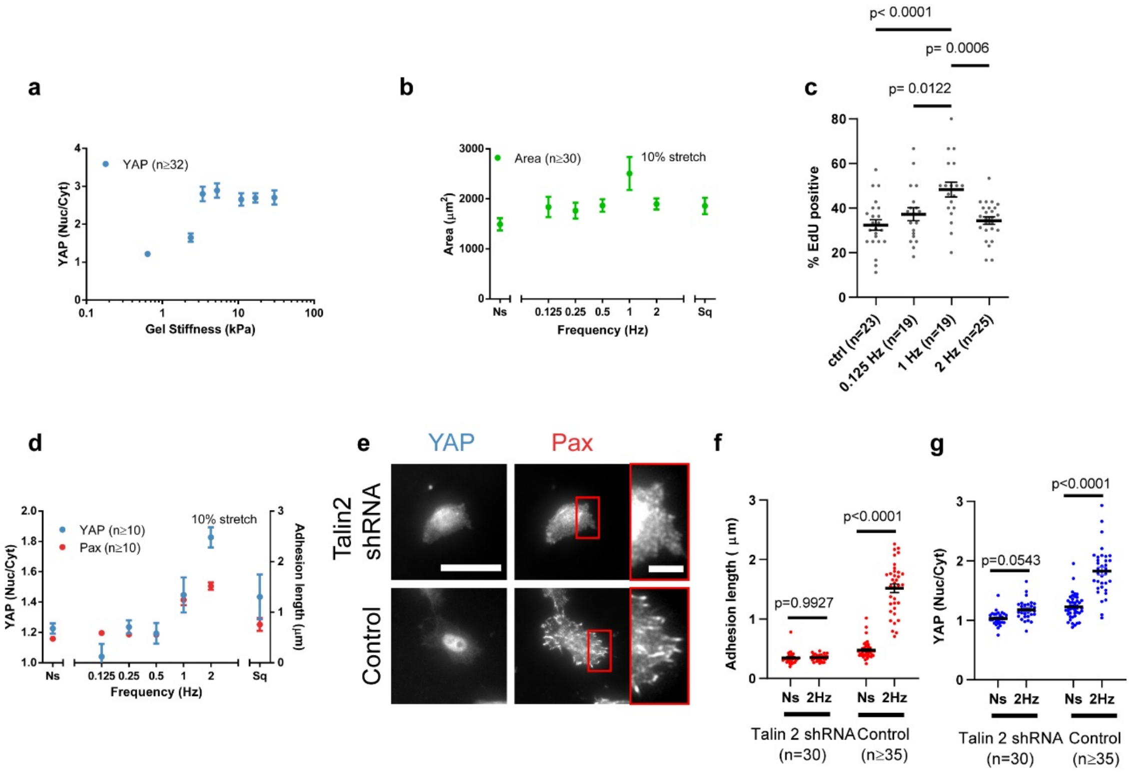

Supplementary Figure 1: Additional information on stretch experiments. a, For mouse embryonic fibroblasts (cells used throughout the paper), quantification of YAP nuclear to cytoplasmic ratios for control cells plated on gels of increasing rigidity. The effect of stiffness was significant (p<0.0001, Kruskal-Wallis test). **b**, Quantification of cell area for cells stretched 10%. The effect of frequency was significant (p=0.0085, Kruskal-Wallis test). **c** For cells stretched by 10%, quantification of cell proliferation rates as a function of stretch frequency, as assessed with an EdU incorporation assay (one-way ANOVA). **d,** Quantification of YAP nuclear to cytoplasmic ratios and focal adhesion lengths for Talin 1 knockdown (control) cells stretched at 10%. These cells overexpress talin 2 and have a wild-type phenotype[20] , and are the control for subsequent talin 2 depletion. The effects of frequency were significant for both YAP and paxillin (p<0.0001). The effect of square versus triangular 1 Hz signals was significant only for paxillin (p=0.0042). Statistical significance was assessed with Kruskal-Wallis test. **e,** YAP and paxillin stainings of Talin 1 knockdown (control) cells, and Talin 2 shRNA cells stretched at 10%, 2 Hz. In the paxillin image, areas circled in red are shown magnified at the right. These cells showed the peak of response at 2 Hz, and subsequent comparisons were therefore carried out at this frequency. Scale bar is 50 µm in main image ,10 µm in magnifications. **f,g** Quantifications of focal adhesion lengths (f) and YAP nuclear to cytoplasmic ratios (g) for Talin 1 knockdown (control) cells, and Talin 2 shRNA cells either not stretched (Ns) or stretched at 10% with a frequency of 2 Hz. Scale bar is 30 µm. n numbers are cells in all panels. Statistical significance was assessed with 2-way ANOVA. Data are shown as mean ± s.e.m.

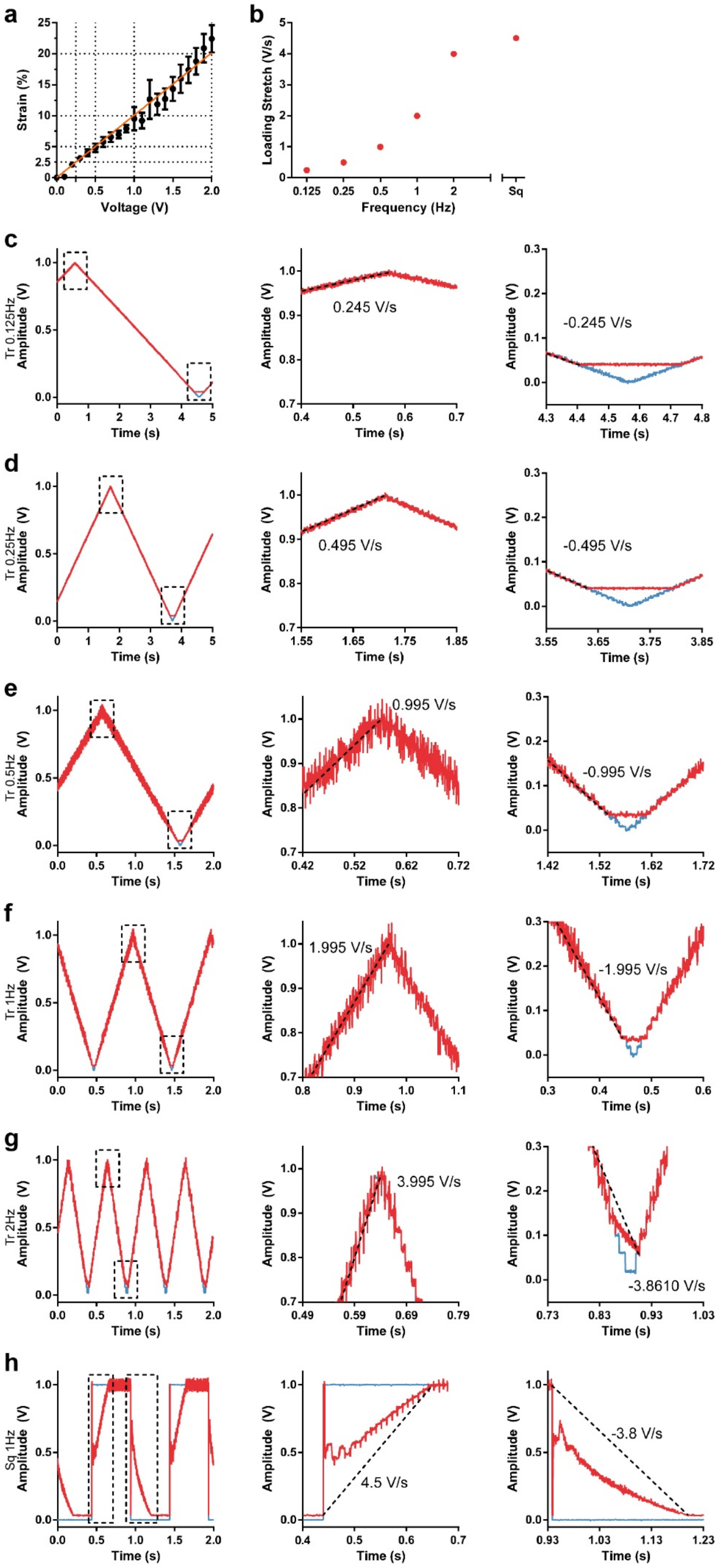
a
Strain (%)
Voltage (V)
b
Loading Stretch (V/s)
Frequency (Hz)
0.125
0.25
0.5
1
2
Sq
c
Tr 0.125Hz Amplitude (V)
Amplitude (V)
Time (s)
0.245 V/s
-0.245 V/s
d
Tr 0.25Hz Amplitude (V)
0.495 V/s
-0.495 V/s
e
Tr 0.5Hz Amplitude (V)
0.995 V/s
-0.995 V/s
f
Tr 1Hz Amplitude (V)
1.995 V/s
-1.995 V/s
g
Tr 2Hz Amplitude (V)
3.995 V/s
-3.8610 V/s
h
Sq 1Hz Amplitude (V)
4.5 V/s
-3.8 V/s

Supplementary Figure 2: Additional characterization of the cell stretch setup. a, Calibration of measured gel stretch as a function of the voltage applied on the pressure transducer (n=3 membranes). **b**, Applied stretch rate as a function of frequency applied. **c-h,** Example traces of applied and measured voltage signals at the pressure transducer for triangular signals at 0.125 Hz (c), 0.25 Hz (d) 0.5 Hz (e), 1 Hz (f), and 2 Hz (g), and square signals at 1Hz (h). Data are shown as mean ± s.e.m.

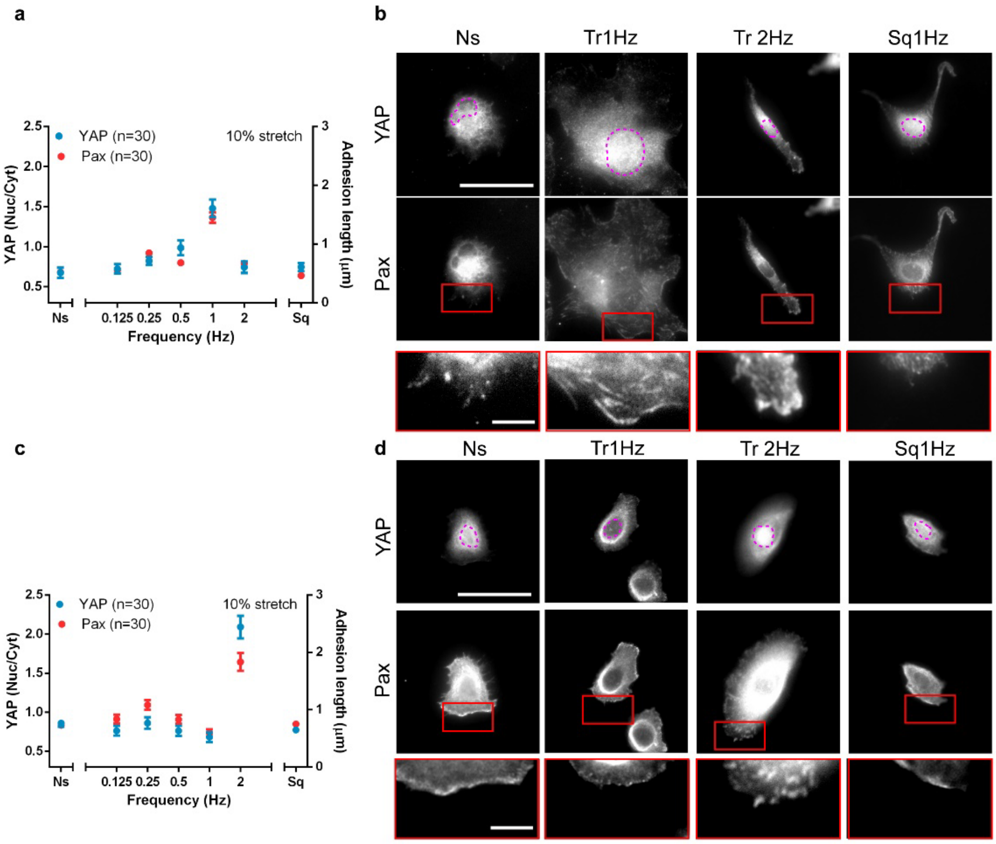

Supplementary Figure 3: Response to stretch of lung endothelial and epithelial cells. a, quantifications of YAP nuclear to cytoplasmic ratios and paxillin focal adhesion lengths in human microvascular endothelial cells (HMVEC) stretched by 10%. Results are shown for non-stretched cells (Ns), cells stretched with triangular signals at different frequencies, and cells stretched with a square signal at 1 Hz (Sq). n numbers are cells. The effects of frequency were significant for both YAP and paxillin (p<0.0001). The effect of square versus triangular 1 Hz signals was significant both YAP and paxillin (p<0.0001). Statistical significance was assessed with 2-way ANOVA. **b**, Corresponding examples of YAP and paxillin stainings. In YAP images, magenta outline indicates the nucleus. In the paxillin image, areas circled in red are shown magnified at the botttom. **c**,**d** Same information as in a,b for small airway epithelial cells (SAEC). The effects of frequency were significant for both YAP and paxillin (p<0.0001). The effect of square versus triangular 1 Hz signals was not significant for either YAP or paxillin (p<0.0001). n numbers are cells. Statistical significance was assessed with 2-way ANOVA. Scale bars are 40 µm. Data are shown as mean ± s.e.m.

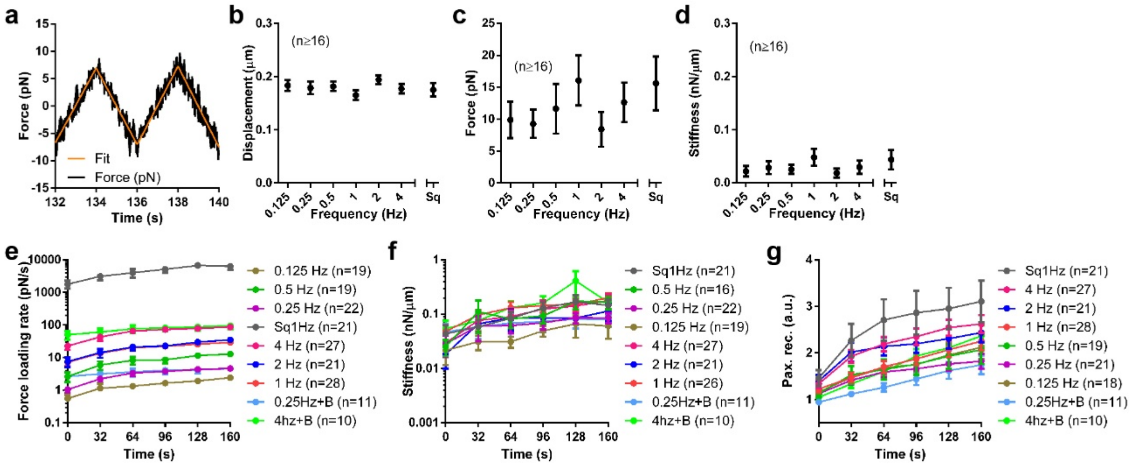

Supplementary Figure 4: Additional results of the optical tweezers experiments. a, Example trace and fit of a measured force signal. To calculate force loading rates, the slope of the fitted lines was taken. **b,** Bead displacement amplitude at time 0s for all frequencies. The effect of frequency was not significant. **c,** Bead force amplitude at time 0s for all frequencies. The effect of frequency was not significant. **d,** Bead stiffness at time 0s for all frequencies. The effect of frequency was not significant. **e,** Force loading rate as a function of time for all conditions. **f,** Stiffness as a function of time for all conditions. **g,** Recruitment of GFP-paxillin to beads as a function of time for all conditions. N numbers are beads in all panels. Data are shown as mean ± s.e.m.

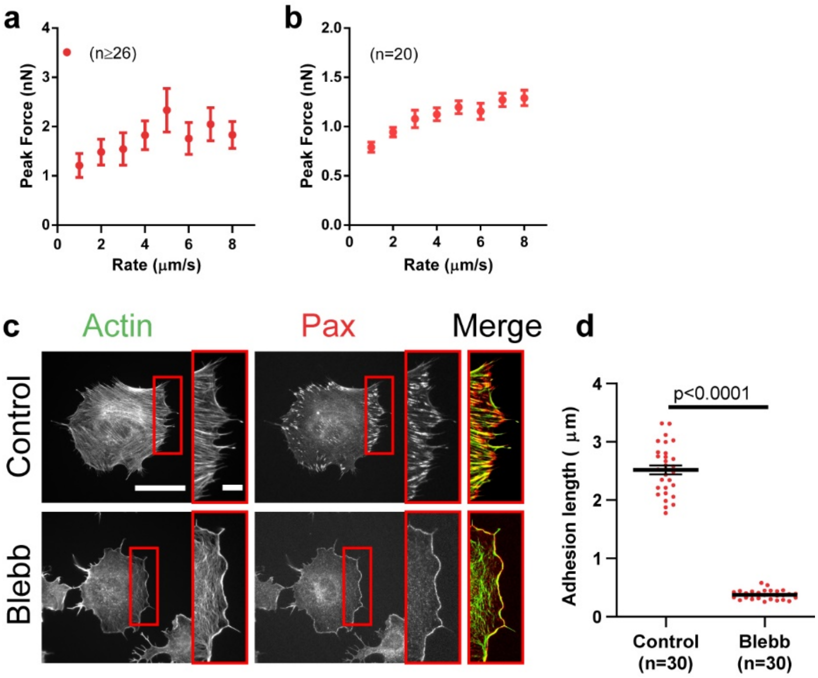

Supplementary Figure 5: Additional AFM and blebbistatin data. **a**, Peak force during cantilever retraction as a function of the retraction speed for cells attaching to a fibronectin-coated substrate. The effect of retraction speed was significant (p<0.0001, Friedman test). n numbers are curves. **b,** Peak force during cantilever retraction as a function of the retraction speed for fibronectin-coated beads attaching to cells. The effect of retraction speed was significant (p<0.0001, friedman test). n numbers are curves. **c**, Actin and paxillin stainings in control cells seeded on glass with or without Blebbistatin treatment. Areas circled in red are shown magnified at the right of each image, and shown as a merged image (actin, green, paxillin, red). Blebbistatin disrupts actin stress fibres but not lamellar actin. Scale bar is 50 µm in main images, 10 µm in magnifications. **d**, Quantification of the adhesion length for control cells, and cells treated with blebbistatin. N numbers are cells. Statistical significance was assessed with two-sided t-test. Data are shown as mean ± s.e.m.

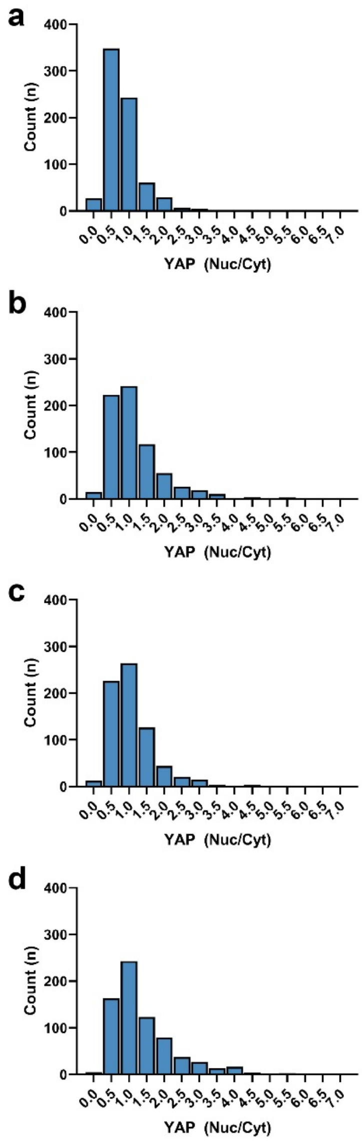

Supplementary Fig. 6: Additional information on rat lung ventilation experiments. Histograms of YAP nuclear to cytoplasmic ratios for rat lung cells ventilated at 0.1 Hz left lung (**a**), 1.1 Hz left lung (**b**), 1.1 Hz right lung (**c**), and 2.1 Hz right lung (**d**). n numbers are cells.

Supplementary tables

Figure	Deformation (μm)	Def. rate (μm/s)	Load (nN)	Loading rate (nN/s)	Load per area (pN/μm^2)	Loading rate per area (pN/μm^2/s)
1 (stretch)	10^{-1} - 10^{0} (i)	10^{-1} - 10^{1}	10^{0} - 10^{1} (ii)	10^{-1} - 10^{1}	10^{0} - 10^{1} (iii)	10^{0} – 10^{2}
3 (optical tweezers)	10^{-1}	10^{-2} - 10^{1}	10^{-2}	10^{-3} - 10^{1}	10^{2} (iv)	10^{0} – 10^{4}
4d (AFM, cells)	10^{0}	10^{0} - 10^{1}	10^{0}	10^{0}-10^{1}	10^{1} (v)	10^{1} – 10^{3}
4h (AFM, beads)	10^{-1}	10^{0} - 10^{1}	10^{0}	10^{1}-10^{2}	10^{2} (v)	10^{3} – 10^{4}

Supplementary Table 1: Orders of deformations, loads, and rates in the different figures. Since several of the values are approximate estimates, we provide only orders of magnitude for comparison. (i) Deformations were calculated at the cell edge from stretch applied, assuming an average cell radius of 10 μm. (ii) Loading rates calculated from deformation rates taking stiffness values of the order of 2 nN/μm (fig. 3c). (iii) Cell spreading areas were also estimated from an average 10 μm cell radius, considering that adhesions and forces are largely generated the cell edge (estimated to be of ~ 2 μm, maximum size of focal adhesions in our measurements). (iv) Average contact areas between 1 μm beads and cells taken from the literature[80,81]. (v) Contact areas at maximum indentation were estimated to be of ~5 μm^2 (bead-cell contact) and ~25 μm^2 (cell-substrate contact) by using Hertz contact mechanics[82].

% Acrylamide	% Bis-acrylamide	Young's modulus (kPa) (Mean ± s.e.m., N=3 gels, n=30 points)
4	0.03	0.64 ± 0.02
5.5	0.04	2.37 ± 0.04
6.16	0.04	3.40 ± 0.06
7.46	0.04	5.26 ± 0.11
7.5	0.1	10.94 ± 0.15
7.55	0.16	16.80 ± 0.22
12	0.15	29.92 ± 0.31

Supplementary Table. 2: Polyacrylamide gel rigidities measured with AFM

Figure panel	n value
1e	YAP: n=30, 29, 30, 30, 28, 30, 29 cells<br>Paxillin: n=30, 29, 30, 30, 28, 30, 29 cells
1f	YAP: n=50, 29, 30, 30, 30, 30, 30 cells<br>Paxillin: n= 50, 29, 30, 30, 30, 30, 30 cells
1g	YAP: n=40, 30, 30, 30, 30, 30, 30 cells<br>Paxillin: n= 66, 30, 30, 30, 30, 30, 40 cells
1h	YAP: n=38, 30, 30, 30, 29, 30, 30 cells<br>Paxillin: n=38, 30, 30, 30, 29, 30, 30 cells
2b	Control, n =36, 30, 42, 32, Jasp: n= 39, 34.
2c	Control ns: n=66 cells; Control sq: n=40 cells
3h	n=18, 21, 16, 26, 21, 27, 21 beads
3i	n=18, 21, 16, 26, 21, 27, 21 beads
3j	n=18, 21, 16, 26, 21, 27, 21 beads
3k	n=18, 21, 18, 22, 19, 28, 21, 27, 21 beads
4d	n=30, 30, 27, 29, 27, 27, 27, 30 curves
4k	Control ns: n=66 cells; Control 1Hz: n=30 cells
4l	Control ns: n=66 cells; Control 1Hz: n=30 cells
Sup 1a	n=33, 35, 32, 33, 32, 33, 32 cells
Sup 1b	n= 66, 30, 30, 30, 30, 30, 40 cells
Sup 1d	YAP: n=40, 10, 9, 10, 10, 35, 10 cells<br>Paxillin: n=40, 10, 9, 10, 10, 35, 10 cells
Sup 1f	Talin 2 shRNA: n=30, n=30; Control: n=40; n=35
Sup 1g	Talin 2 shRNA: n=30, n=30; Control: n=40; n=35
Sup 2a	n=3 membranes
Sup 4b	n=18, 21, 16, 26, 21, 27, 21 beads
Sup 4c	n=18, 21, 16, 26, 21, 27, 21 beads
Sup 4d	n=18, 21, 16, 26, 21, 27, 21 beads
Sup 5a	n=30, 30, 27, 29, 27, 27, 27, 30 curves

Supplementary Table 3: Exact n values for panels where the information did not fit in the figure.

Parameter	meaning	Value	Origin
n_f	Number of FN molecules	1200	[20]
K_{oni}	Intrinsic binding rate	$2.11x10^{-4}um^2/s$	[20]
K_{off}	Integrin unbinding rate	Catch bond	[20]
K_{fold}	Talin unfolding rate	Slip bond	[20]
k_c	Clutch spring constant	1 nN/nm	[20]
FR	Fraction of force experienced by talin	0.073	[20]
k_{sub}	Substrate spring constant	1 µN/nm	Lower range in[20]
d_{min}	Minimum (baseline) Integrin density on the membrane	300 /µm^2	[20]
d_{max}	Maximum integrin density on the membrane	1200 /µm^2	Adjusted
d_{add}	Integrins added after each reinforcement event	24 /µm^2	Adjusted
A	Amplitude of stretch signal	1.5 – 3 µm	Adjusted
fr	Frequency of stretch signal	0.125 Hz – 2 Hz	Experimental values
F_{act}	Force for cytoskeletal softening	142 pN	Adjusted

Supplementary table 4. Model parameters
