# Supplementary material for: The force loading rate drives cell mechanosensing through both reinforcement and cytoskeletal softening
Source: Nat Commun. 2021 Jul 9;12:4229. doi: 10.1038/s41467-021-24383-3 (PMC8270983; doi:10.1038/s41467-021-24383-3)
Supplement: Supplementary file 3 — Description of Additional Supplementary Files [file 41467_2021_24383_MOESM3_ESM.pdf]

Description of Additional Supplementary Files

File Name: Supplementary Movie 1
Description: Time lapse of cell transfected with LifeAct-GFP and plated on a polyacrylamide gel of 0.6 kPa in stiffness.

File Name: Supplementary Movie 2
Description: Time lapse of cell transfected with LifeAct-GFP and plated on fibronectin-coated glass.

File Name: Supplementary Software 1
Description: Codes employed for the computational clutch model, and to analyze AFM and optical tweezers data.
