# Supplementary material for: The force loading rate drives cell mechanosensing through both reinforcement and cytoskeletal softening
Source: Nat Commun. 2021 Jul 9;12:4229. doi: 10.1038/s41467-021-24383-3 (PMC8270983; doi:10.1038/s41467-021-24383-3)
Supplement: Supplementary file 6 — Supplementary Software 1 [file 41467_2021_24383_MOESM6_ESM.zip › README.docx]

**Codes for AFM Data**

Source code:

MATLAB Code to analyse jpkforce files using MATLAB 2019a, and to extract stiffness from curves.

System requirements:

Software dependencies

MATLAB 2019a (tested on)

Operating Systems

Windows 10

Windows 7 Service Pack 1

Windows Server 2019

Windows Server 2016

Processors

Minimum: Any Intel or AMD x86-64 processor

Disk

Minimum: 3.1 GB of HDD space for MATLAB only, 5-8 GB for a typical installation

RAM

Minimum: 4 GB

Graphics

No specific graphics card is required.

A full installation of all MathWorks products may take up to 31 GB of disk space (for more info: <https://www.mathworks.com/support/requirements/matlab-system-requirements.html>)

Instructions for use:

This code is written for batch processing of the curves.

First have the JPKFORCE files in a folder ready then use afmDataAnalyser.m file.

At last StiffnessAndLoadingRateCalculator.m for calculating stiffness and loading rate.

Demo:

“Sample data” folder has a demo file.

Run afmDataAnalyser.m. Select the sample data folder


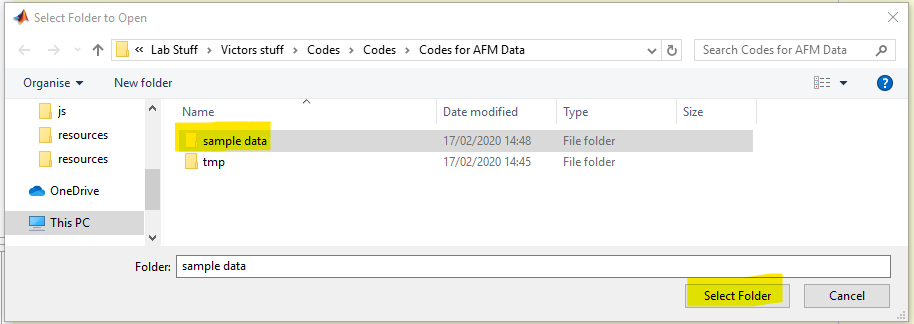

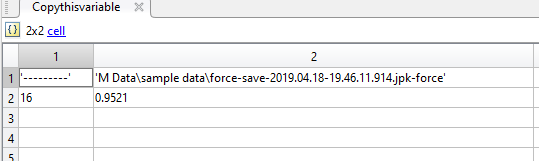


Code runs and finishes with all the data stored in variable in the workspace called “Copythisvariable”

It contains two columns:

Frequency and Force

**
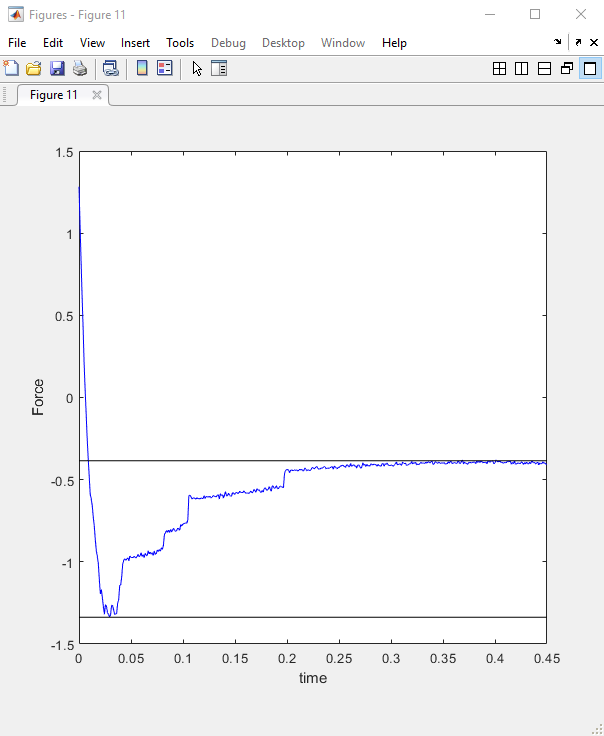
**And the code plots for the force time curve is plotted

Run StiffnessAndLoadingRateCalculator.m. Select the sample data folder


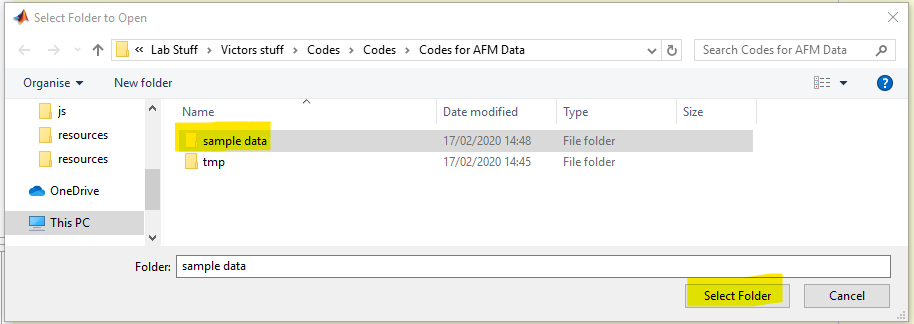


Then you get another “copythisvariable”

With columns for Frequency and loading rate

Frequency and Stiffness

There are two stiffness reported, stiffness obtained by linear fit, and stiffness obtained by contact curve fitting.


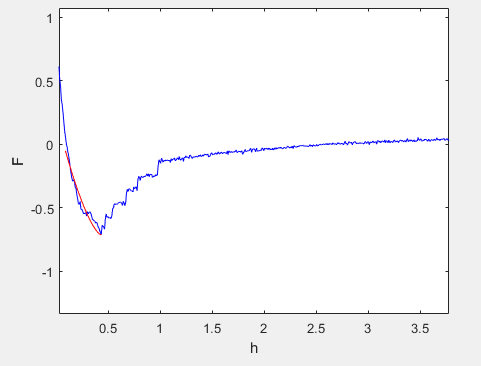


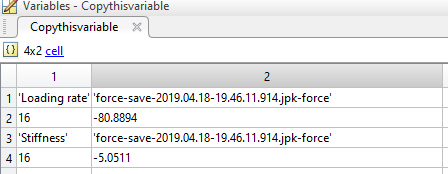


It takes a minute to run for 10 curves

**Codes for Optical Tweezers (OT) Data**

Source code:

MATLAB Code to analyse OT Data files using MATLAB 2019a for calculating loading and strain rates.

System requirements:

Software dependencies

MATLAB 2019a (tested on)

Operating Systems

Windows 10

Windows 7 Service Pack 1

Windows Server 2019

Windows Server 2016

Processors

Minimum: Any Intel or AMD x86-64 processor

Disk

Minimum: 3.1 GB of HDD space for MATLAB only, 5-8 GB for a typical installation

RAM

Minimum: 4 GB

Graphics

No specific graphics card is required.

A full installation of all MathWorks products may take up to 31 GB of disk space (for more info: <https://www.mathworks.com/support/requirements/matlab-system-requirements.html>)

Instructions for use:

This code is written for batch processing of the folders.

Get all the data in a directory "X"

Create a list according to name, folder, frequency,date

Run the AnalysingAllTheDataFromServer.m

Demo:

Run AnalysingAllTheDataFromServer.m. Select the response_function_set_1017 folder

Code runs and finishes with all the data stored in variable in the workspace called “Data”

It contains two columns:


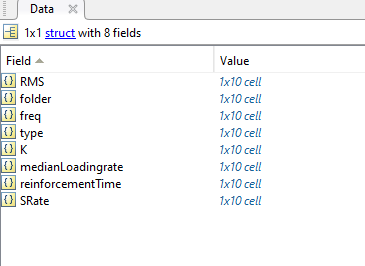
Parameter and values

Folder name

Frequency

Type of data

Median loading rate

Reinforcement time

Strain rates

It takes 3 seconds per data points for running.

**Code for computational clutch model.**

Source code:

MATLAB Code to run computational clutch model as described in the manuscript

System requirements:

Software dependencies

MATLAB 2019a (tested on)

Operating Systems

Windows 10

Windows 7 Service Pack 1

Windows Server 2019

Windows Server 2016

Processors

Minimum: Any Intel or AMD x86-64 processor

Disk

Minimum: 3.1 GB of HDD space for MATLAB only, 5-8 GB for a typical installation

RAM

Minimum: 4 GB

Graphics

No specific graphics card is required.

A full installation of all MathWorks products may take up to 31 GB of disk space (for more info: <https://www.mathworks.com/support/requirements/matlab-system-requirements.html>)

Instructions for use:

The actual model is in file cmstretchg.m. A script (stretchscriptg.m) is also provided, which can be used to call the model with the range of parameters used in the manuscript. The script also compares the model to experimental data, provided in file “experimental data.mat”.

The script called “loading rate script.m” can be used to calculate a matrix of how a specific event (integrin unbinding or talin unfolding) depends on both a starting force F and a constant applied loading rate L. Values for the matrices used in simulations are also provided in file kofflr.mat. Please note that the script was only used to calculate the rates of integrin unbinding and talin unfolding events. For actin filament breakage (fluidization), instantaneous breakage was assumed above a threshold force, thus the matrix has values of zero below that force, and infinity above (regardless of loading rate). Talin refolding was not considered, and thus a rate of zero was assumed.
