# Supplementary figures and images for: The force loading rate drives cell mechanosensing through both reinforcement and cytoskeletal softening
Source: Nat Commun. 2021 Jul 9;12:4229. doi: 10.1038/s41467-021-24383-3 (PMC8270983; doi:10.1038/s41467-021-24383-3)

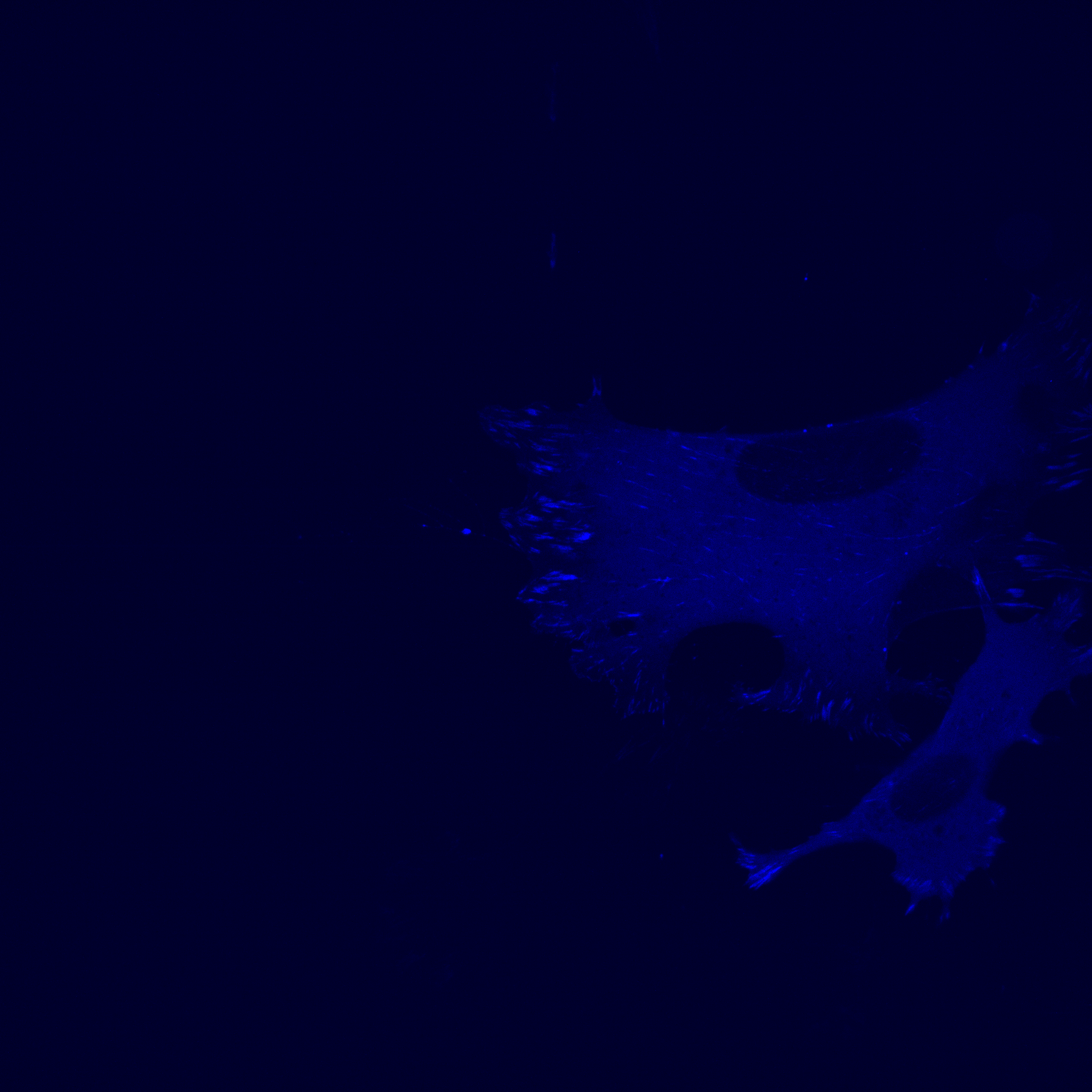

Supplement: Supplementary file 6 — Supplementary Software 1 [file 41467_2021_24383_MOESM6_ESM.zip › Codes for OT Data/response_function_set_1017/calibration_end_image.png]

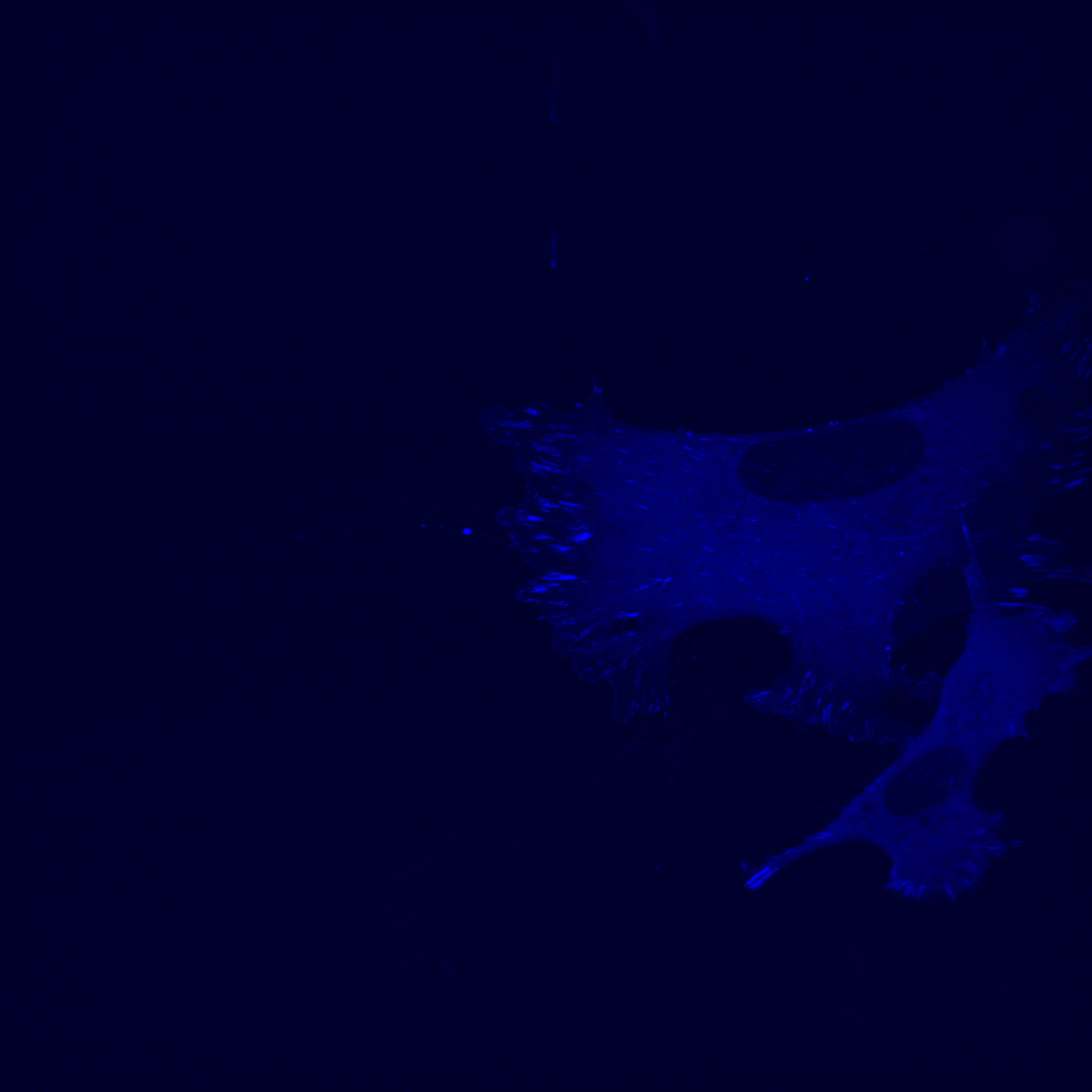

Supplement: Supplementary file 6 — Supplementary Software 1 [file 41467_2021_24383_MOESM6_ESM.zip › Codes for OT Data/response_function_set_1017/calibration1000_image.png]
