# Supplementary material for: The force loading rate drives cell mechanosensing through both reinforcement and cytoskeletal softening
Source: Nat Commun. 2021 Jul 9;12:4229. doi: 10.1038/s41467-021-24383-3 (PMC8270983; doi:10.1038/s41467-021-24383-3)
Supplement: Supplementary file 7 — Reporting Summary [file 41467_2021_24383_MOESM7_ESM.pdf]

nature research

Corresponding author(s): Pere Roca-Cusachs

Last updated by author(s): Apr 23, 2021

# Reporting Summary

Nature Research wishes to improve the reproducibility of the work that we publish. This form provides structure for consistency and transparency in reporting. For further information on Nature Research policies, see Authors & Referees and the Editorial Policy Checklist .

## Statistics

For all statistical analyses, confirm that the following items are present in the figure legend, table legend, main text, or Methods section.

| n/a | Confirmed | |
|---|---|---|
| ☐ | ☒ | The exact sample size (*n*) for each experimental group/condition, given as a discrete number and unit of measurement |
| ☐ | ☒ | A statement on whether measurements were taken from distinct samples or whether the same sample was measured repeatedly |
| ☐ | ☒ | The statistical test(s) used AND whether they are one- or two-sided<br>*Only common tests should be described solely by name; describe more complex techniques in the Methods section.* |
| ☒ | ☐ | A description of all covariates tested |
| ☐ | ☒ | A description of any assumptions or corrections, such as tests of normality and adjustment for multiple comparisons |
| ☐ | ☒ | A full description of the statistical parameters including central tendency (e.g. means) or other basic estimates (e.g. regression coefficient) AND variation (e.g. standard deviation) or associated estimates of uncertainty (e.g. confidence intervals) |
| ☐ | ☒ | For null hypothesis testing, the test statistic (e.g. *F*, *t*, *r*) with confidence intervals, effect sizes, degrees of freedom and *P* value noted<br>*Give P values as exact values whenever suitable.* |
| ☒ | ☐ | For Bayesian analysis, information on the choice of priors and Markov chain Monte Carlo settings |
| ☒ | ☐ | For hierarchical and complex designs, identification of the appropriate level for tests and full reporting of outcomes |
| ☒ | ☐ | Estimates of effect sizes (e.g. Cohen's *d*, Pearson's *r*), indicating how they were calculated |

*Our web collection on statistics for biologists contains articles on many of the points above.*

## Software and code

Policy information about availability of computer code

| | |
|---|---|
| Data collection | Inmunostainings were imaged by using Metamorph (Version 7.7.10) or Micromanager (version 1.4.22). Optical tweezers data acquired using a custom LabVIEW software (2014 Service Pack 1 Version 14.0.1f7). AFM data was acquired using the JPK software (JPK Data Processing Version 6.1.79). |
| Data analysis | Inmunostaining images were analyzed with ImageJ (version 1.53e). Data statistic tests were performed with Graphpad PRISM (version 9). Optical tweezers and AFM data were analysed using custom matlab scripts (Matlab R2020a). Actin anisotropy was analyzed using FibrilTool ImageJ plugin. The computational clutch model was implemented in Matlab (version R2020a). |

For manuscripts utilizing custom algorithms or software that are central to the research but not yet described in published literature, software must be made available to editors/reviewers. We strongly encourage code deposition in a community repository (e.g. GitHub). See the Nature Research guidelines for submitting code & software for further information.

## Data

Policy information about availability of data

All manuscripts must include a data availability statement. This statement should provide the following information, where applicable:

- Accession codes, unique identifiers, or web links for publicly available datasets
- A list of figures that have associated raw data
- A description of any restrictions on data availability

The authors declare that all data supporting the findings of this study are available within the paper and its supplementary information files.

# Field-specific reporting

Please select the one below that is the best fit for your research. If you are not sure, read the appropriate sections before making your selection.

☒ Life sciences ☐ Behavioural & social sciences ☐ Ecological, evolutionary & environmental sciences

For a reference copy of the document with all sections, see nature.com/documents/nr-reporting-summary-flat.pdf

# Life sciences study design

All studies must disclose on these points even when the disclosure is negative.

| | |
|---|---|
| Sample size | No statistical methods were used to determine sample size before execution of the experiments. Sample sizes for number of cells were determined based on previous experience by our group and others on similar experiments. |
| Data exclusions | No data was excluded from the analyses. |
| Replication | All experimental findings were reproduced independently at least two times. |
| Randomization | Cells and animals were measured at random within each condition. |
| Blinding | Due to high in vivo variability, investigators were blinded during rat lung immunostainings experiments and analysis. In other experiments, blinding was not considered to be necessary due to the clear effects observed. |

# Reporting for specific materials, systems and methods

We require information from authors about some types of materials, experimental systems and methods used in many studies. Here, indicate whether each material, system or method listed is relevant to your study. If you are not sure if a list item applies to your research, read the appropriate section before selecting a response.

## Materials & experimental systems

| n/a | Involved in the study | |
|---|---|---|
| ☐ | ☒ | Antibodies |
| ☐ | ☒ | Eukaryotic cell lines |
| ☒ | ☐ | Palaeontology |
| ☐ | ☒ | Animals and other organisms |
| ☒ | ☐ | Human research participants |
| ☒ | ☐ | Clinical data |

## Methods

| n/a | Involved in the study | |
|---|---|---|
| ☒ | ☐ | ChIP-seq |
| ☒ | ☐ | Flow cytometry |
| ☒ | ☐ | MRI-based neuroimaging |

## Antibodies

| | |
|---|---|
| Antibodies used | Primary antibodies used were anti-Paxillin rabbit clonal (Y113, abcam, ab32084), and anti-YAP mouse monoclonal (63.7, Santa Cruz Biotechnology, sc-101199), 1:200. Secondary antibodies used were Alexa Fluor 488 anti-mouse (A-11029, Thermo Fischer Scientific), Alexa Fluor 488 anti-rabbit (A-21206; Thermo Fischer Scientific), Alexa Fluor 555 anti-mouse (A-21422; Thermo Fischer Scientific)and Alexa Fluor 555 anti-rabbit (A-21429, Thermo Fischer Scientific) 1:500. We used phalloidin (Alexa Fluor 555 phalloidin, Thermo Fischer Scientific) 1:1000, and Hoechst (33342, Thermo Fischer Scientific) 1:2000. |
| Validation | YAP and paxillin antibodies are validated for immunofluorescence as described in the manufacturer's webpage. The expected intracellular localization of each antibody further validated them. |

## Eukaryotic cell lines

Policy information about cell lines

| | |
|---|---|
| Cell line source(s) | Male mouse embryonic fibroblasts were described previously (Roca-Cusachs et al., 2013).<br>Talin 1 −/− male mouse embryonic fibroblasts were described previously (Zhang et al., 2008).<br>Primary human small airway epithelial cells (SAEC) were purchased from ATCC.<br>. Primary human lung microvascular endothelial cells (HMVEC) were purchased from Lonza. |
| Authentication | None of the cell lines used were authenticated. |

| | |
|---|---|
| Mycoplasma contamination | Cell cultures were routinely checked for mycoplasma. Cell lines tested negative for mycoplasma contamination. |
| Commonly misidentified lines (See ICLAC register) | No commonly misidentified lines were used. |

## Animals and other organisms

Policy information about studies involving animals; ARRIVE guidelines recommended for reporting animal research

| | |
|---|---|
| Laboratory animals | Twelve pathogen-free male Sprague Dawley rats (350-450 g) were randomly distributed in the different experimental groups. Animals were housed in controlled animal quarters under standard light, temperature and humidity exposure. |
| Wild animals | No wild animals were used |
| Field-collected samples | No field-collected samples were used |
| Ethics oversight | All experimental procedures were approved by the Ethical Committee for Animal Research of the University of Barcelona (Approval number 147/18). |

Note that full information on the approval of the study protocol must also be provided in the manuscript.
